# Interaction between alcohol consumption and methylenetetrahydrofolate reductase polymorphisms in thyroid cancer risk: National Cancer Center cohort in Korea

**DOI:** 10.1038/s41598-018-22189-w

**Published:** 2018-03-06

**Authors:** Sarah Yang, Jeonghee Lee, Yoon Park, Eun Kyung Lee, Yul Hwangbo, Junsun Ryu, Joohon Sung, Jeongseon Kim

**Affiliations:** 1 Molecular Epidemiology Branch, Division of Cancer Epidemiology and Prevention, Research Institute, National Cancer Center, Seoul, South Korea; 2 Complex Disease & Genomic Epidemiology Branch, Department of Public Health, School of Public Health, Seoul National University, Seoul, South Korea; 3 Center for Thyroid Cancer, National Cancer Center Hospital, National Cancer Center, Seoul, South Korea

## Abstract

The effect of alcohol intake on thyroid cancer is unestablished, and its interaction effects with genetic susceptibility are unclear. In this case-control study, the relationship among alcohol intake, the methylenetetrahydrofolate reductase (*MTHFR*) gene, and thyroid cancer risk has been evaluated. In total, 642 cases and 642 controls of Korean origin were included, and the genetic variants C677T and A1298C of the *MTHFR* gene were analysed. The interactions between alcohol-consumption behaviour and genetic variants were analysed with a likelihood ratio test, wherein a multiplicative interaction term was added to a logistic regression model. There was an independent association between the C677T polymorphism and thyroid cancer risk but not for drinking history. For C677T C/C homozygotes, individuals with a history of alcohol consumption showed a protective OR (95% CI) of 0.42 (0.15–1.13) when never drinkers were used as the reference. However, this protective association was not observed among individuals with a T+ allele with an OR (95% CI) of 1.27 (0.89–1.82), showing different directions for the association between genotypes with a significant interaction (*P*_interaction_ = 0.009). Based on the genetic characteristics of individuals included, an interaction between alcohol intake and *MTHFR* C677T may modify the risk of thyroid cancer.

## Introduction

Thyroid cancer is a frequent type of endocrine cancer that has continuously increased in recent decades worldwide^1^. The trends for thyroid cancer incidence vary across geographic areas and ethnicities based on the diversity of genetic background, environmental influences, and access to medical care, which may affect controversial issues related to thyroid cancer such as overdiagnosis and overtreatment^1–3^. Thyroid cancer is the most commonly diagnosed cancer in Korea, with an age-standardized incidence rate of 60.1 per 100,000 (adjusted using the world standard population), which is the highest among all cancers^4^. The causes for this high incidence rate of thyroid cancer in Korea may be attributed by overdiagnosis^5^. However, overdiagnosis alone does not fully explain either the non-proportionally distributed incidence across age and sex or the observed birth cohort patterns^1^. There are potential carcinogenic factors that may contribute to the increased incidence of thyroid cancer, although the specific mechanisms are not fully understood. For instance, exposure to ionizing radiation during medical imaging and nuclear medicine procedures is a well-known exogenous risk factor for thyroid cancer. Iodine intake and environmental pollutants may also affect endogenous mechanisms that control hormonal release and oxidative stress in the thyroid^1,6^. Among the modifiable risk factors related to cancer, including tobacco use and dietary habits, alcohol consumption is a well-known avoidable risk factor for many types of cancer, and the International Agency for Research on Cancer (IARC) has classified ethanol in alcoholic beverages as a Group 1 carcinogen in humans^7–10^. The adverse effect of alcohol on cancer risk has been studied broadly in accordance with folate deficiency, oxidative stress, and carcinogenesis on the molecular and genetic levels^11,12^. Interestingly however, several epidemiologic studies across diverse populations have suggested that alcohol consumption might have a protective effect on thyroid cancer^13–16^. A meta-analysis by Bagnardi *et al*.^9^ that included 9 independent studies reported the protective odds ratio (OR) for light and moderate drinkers compared to that of non-drinkers. However, there is no established conclusion regarding the thyroid cancer risk and the intake of alcoholic beverages, with no sound biological mechanism to explain the protective association due to discrepancies in the reported relationships and the scarcity of studies. The inverse relationship between alcohol and thyroid cancer may be caused by effect modification by other complex risk factors such as smoking, diet, and genetic characteristics. To explore this inverse trend of risk association, we hypothesized that an individual’s genetic characteristics might affect thyroid cancer risk and alcohol consumption differently. Heavy alcohol intake is known to cause folate deficiency due to intestinal malabsorption, decreased liver uptake, and increased excretion of folic acid via urine^17^. However, in this study, we did not explore the effect of folate itself but rather the genetic susceptibility of the methylenetetrahydrofolate reductase (*MTHFR*) gene, which not only plays a crucial role in folate metabolism affected by alcohol consumption but also is involved in DNA synthesis, the methylation cycle, and epigenetic regulation^18–21^ and is a widely studied one-carbon metabolism-related gene with reported associations with all types of cancer, including thyroid cancer^20,22–24^.

In this case-control study, we examined whether the independent risk association between either alcohol consumption or *MTHFR* genetic variants and thyroid cancer is present in a Korean population. The study also aimed to assess the combined effect of alcohol consumption and *MTHFR* genetic polymorphism on thyroid cancer risk.

## Results

### General characteristics of the study population

General demographic characteristics were compared between the thyroid cancer cases and controls (Table 1). The proportion of individuals with a body mass index (BMI) over 25 was higher among the cases than the controls, while smoking status and education level did not differ between groups. Additionally, the proportion of individuals with a first-degree family history of thyroid cancer was significantly higher among the cases. The alcohol consumption status groups, including never drinkers, ex-drinkers, and current drinkers, showed significant differences based on disease status.

**Table 1:** Characteristics of the subjects according to stratification of disease status^1^.

	Control (*n* = 642)		Case (*n* = 642)		*P* ^2^
	*n*	Mean ± SD	*n*	Mean ± SD	
Age	642	48.55 ± 8.47	642	48.55 ± 8.47	>0.999
Sex [*n* (%)]					
Male		201(31.31)		201(31.31)	>0.999
Female		441(68.69)		441(68.69)	
Body mass index (kg/m^2^) [*n* (%)]					0.007
<23		297(46.62)		243(37.91)	
23~25		142(22.29)		167(26.05)	
≥25		198(31.08)		231(36.04)	
Missing		5		1	
Education [*n* (%)]					0.315
Elementary		43(6.70)		48(7.48)	
Middle-high		308(47.98)		281(43.77)	
University		291(45.33)		313(48.75)	
1st degree family history of thyroid cancer [*n* (%)]					
No		628(98.13)		599(93.59)	<0.001
Yes		12(1.88)		41(6.41)	
Missing		2		2	
Smoking status [*n* (%)]					
Non-smoker		421(66.83)		445(70.30)	0.413
Ex-smoker		96(15.24)		86(13.59)	
Current smoker		113(17.94)		102(16.11)	
Missing		12		9	
Alcohol consumption status [*n* (%)]					
Non-drinker		263(40.97)		289(45.02)	<0.001
Ex-drinker		18(2.80)		45(7.01)	
Current drinker		361(56.23)		308(47.98)	

^1^SD standard deviation.

^2^Significant difference between case and control obtained by Student’s t-test and the chi-squared test.

### Independent associations of thyroid cancer risk with alcohol consumption history and *MTHFR* polymorphisms

When the participants were classified as never or ever drinkers, alcohol consumption status was not significantly associated with thyroid cancer risk, although the OR estimate for ever drinkers compared to that of never drinkers suggested an inverse association (fully adjusted OR, 0.86; 95% confidence interval (CI), 0.67–1.11) (Table 2). Additionally, no significant association was observed when stratified by sex. When the groups were stratified by average intake, light drinkers (<4.7 g of alcohol/day) showed a significant OR of 0.71 (95% CI, 0.51–0.98) compared to never drinkers, while moderate/heavy drinkers (≥4.7 g/day) showed an insignificant association. Moreover, this association was apparent only in moderate/heavy drinking females when stratified by sex, likely because there were more study subjects available in this group (Supplemental Table 1).

**Table 2:** Association between alcohol consumption and thyroid cancer risk, stratified by sex^1^.

	Control	Case	Crude OR (95% CI)	Fully adjusted OR (95% CI)^2^
**Alcohol consumption status [** ***n*** **(%)]**				
Total (*n* = 1,284)				
Never drinker	263(40.97)	289(45.02)	1.00	1.00
Ever drinker	379(59.03)	353(54.98)	0.83(0.65–1.05)	0.86(0.67–1.11)
Males (*n* = 402)				
Never drinker	24(11.94)	32(15.92)	1.00	1.00
Ever drinker	177(88.06)	169(84.08)	0.74(0.43–1.27)	0.80(0.46–1.41)
Females (*n* = 882)				
Never drinker	239(54.20)	257(58.28)	1.00	1.00
Ever drinker	202(45.80)	184(41.72)	0.85(0.65–1.11)	0.88(0.67–1.17)

^1^OR, odds ratio; CI, confidence interval; estimates of the ORs and 95% CIs were all rounded to the nearest tenth.

^2^Adjusted by body mass index, smoking status, education level, and 1^st^ degree family history of thyroid cancer.

The MAFs of A1298C and C677T were 0.17 and 0.46, respectively. The pair wise linkage disequilibrium (R^2^) between C677T and A1298C was 0.17 (Supplemental Table 2). The effect of each genetic variant was analysed using the dominant model. Compared to A/A homozygotes of A1298C, individuals with minor allele C showed an OR of 0.80 (95% CI, 0.64–1.01) for thyroid cancer risk. By contrast, individuals with the C677T T+ genotype showed a significantly increased risk (OR, 1.37; 95% CI, 1.08–1.75) compared to individuals with the C/C genotype (Table 3).

**Table 3:** Association between *MTHFR* variants and thyroid cancer risk in the dominant model^1^.

	Control/Case	Odds ratio (95% CI)	Chi-square *P*-value
A1298C genotype			
A/A	431/463	1.00	0.05
C+	211/179	0.80 (0.64–1.01)	
C677T genotype			
C/C	211/169	1.00	0.01
T+	431/473	1.37 (1.08–1.75)	

^1^CI, confidence interval; Estimates of the odds ratios and 95% CIs were all rounded to the nearest tenth.

### Interaction between alcohol exposure and *MTHFR* variants in thyroid cancer risk

Stratification by alcohol consumption status and *MTHFR* variants in the dominant model was performed to analyse their interacting effect in association with thyroid cancer risk (Table 4). Compared to never drinkers, ever drinkers with the A/A genotype of A1298C had an insignificant OR of 0.94(95% CI, 0.79–1.76) while individuals with the C+ allele showed an OR of 0.56(95% CI, 0.20–1.60) (*P* for interaction = 0.559). For C677T C/C homozygotes, individuals with an alcohol consumption history showed a protective OR estimate of 0.42 (95% CI, 0.15–1.13) when never drinkers were used as the reference. However, individuals with a T+ allele of C677T showed an insignificant association with thyroid cancer (OR, 1.27; 95% CI, 0.89–1.82), indicating the opposite trend in the association’s direction between different genotypes with a significant interaction (*P* for interaction = 0.009). When the interaction between alcohol consumption dose and *MTHFR* variants was evaluated to observe any dose-response effect, no significant interaction was observed (data not shown).

**Table 4:** Interaction of alcohol consumption status and *MTHFR* variants (dominant model) with thyroid cancer risk^1^.

Genotype	Alcohol consumption status	Control/Case	Crude OR (95% CI)	Fully adjusted OR (95% CI)^2^	*P* for interaction^2^
A1298C					
A/A	Never	182/210	1.00	1.00	0.559
	Ever	249/253	0.86(0.61–1.21)	0.94(0.65–1.37)	
C+	Never	81/79	1.00	1.00	
	Ever	130/100	0.50(0.19–1.33)	0.56(0.20–1.60)	
C677T					
C/C	Never	78/85	1.00	1.00	0.009
	Ever	133/84	0.40(0.16–1.03)	0.42(0.15–1.13)	
T+	Never	185/204	1.00	1.00	
	Ever	246/269	1.17(0.84–1.64)	1.27(0.89–1.82)	

^1^OR, odds ratio; CI, confidence interval; estimates of the ORs and 95% CIs were all rounded to the nearest tenth.

^2^Adjusted by body mass index, smoking status, education, and 1^st^ degree family history of thyroid cancer.

## Discussion

Our findings demonstrated that a history of alcohol consumption may reduce thyroid cancer risk with limited evidence, and there was an independent association between the *MTHFR* genetic polymorphism C677T and thyroid cancer risk. C/C monozygotes of *MTHFR* C677T with a history of drinking alcohol showed a decreased risk of thyroid cancer, where an increasing trend was observed in individuals with a minor allele T. These associations support the importance of considering alcohol consumption and alcohol-related genetic variations when defining groups at high risk for thyroid cancer in a Korean population.

Higher alcohol intake is a global modifiable risk factor for cancer^25^ that affects numerous organs and systems of the human body, including impaired folate metabolism, vitamin deficiency, oxidative stress and DNA damage, which may lead to carcinogenesis^26^. A recent study conducted in Korea reported that threshold effects of acute high-dose and long-term alcohol consumption are associated with an increase in thyroid cancer^27^. However, several prospective studies examining the risk association between alcohol use and thyroid cancer have consistently shown a clear reduction in the risk of thyroid cancer with higher alcohol consumption^15,16,28–30^, supporting the results of our study. Previous studies have indicated that alcohol may affect thyroid cancer development differently by inhibiting the thyroid-specific hormonal response of elevated thyroid-stimulating hormone (TSH), which is related to increased thyroid cell proliferation, although its effect is not clear in humans^13,31^. Further studies are required to explore the negative association between alcohol and thyroid cancer risk.

The critical role that DNA methylation plays in gene regulation has been widely studied and implicated in the risk of developing chronic diseases including cancer^32,33^. The *MTHFR* gene is known to be involved in folate metabolism, playing an essential role in inherited DNA methylation profiles, and *MTHFR* C677T is a common polymorphism encoding an enzyme whose activity level is affected by the levels of folate^34^. Additionally, it has been reported that individuals with the TT genotype of *MTHFR* C677T have only approximately 30% of the *MTHFR* enzyme activity compared to CC homozygotes and 65% of the activity for CT^35^. There are few previous studies assessing the association between *MTHFR* C677T and thyroid cancer, and the reported findings are inconsistent. According to a case-control study that examined the association between germ-line polymorphism in the *MTHFR* gene and differentiated thyroid carcinoma (DTC), a 2.33-fold increased risk was observed for DTC in individuals with the 677TT genotype compared to the controls^20^. However, no significant associations between papillary thyroid cancer (PTC) and the T/T genotype of *MTHFR* C677T were reported in a previous Korean study, showing some discrepancies between different anatomical sites of thyroid carcinoma^36^. The findings of the present study support the presence of a minor allele T being associated with a 1.37-fold increased risk of thyroid cancer. Further replicated large-scale studies are necessary to validate the association between *MTHFR* C677T and thyroid cancer.

Genetic susceptibility that may account for numerous metabolic reactions, including activation or deactivation of metabolites and DNA repair, is affected by other susceptibility factors, such as demographics and nutritional status^37^. A significant interaction between history of alcohol consumption and the C677T polymorphism affecting thyroid cancer risk was observed in this study. It has been suggested that the cancer risk associated with *MTHFR* polymorphisms may be modulated by alcohol intake, but only a few previous studies have examined this issue in detail regarding thyroid cancer risk. A similar Brazilian study was recently conducted to determine the interaction between alcohol consumption and *MTHFR* C677T on thyroid cancer risk, but the interactive effect was not statistically significant (*P* for interaction = 0.84)^38^. The inconsistency between the Brazilian study and the current study may be due to differences in genetic characteristics of the studied populations, which may affect certain biochemical reactions after alcohol consumption; for instance, the *ALDH2* genetic variant is prevalent only in East Asians and is associated with an inability to metabolize a carcinogenic alcohol metabolite, acetaldehyde, into acetate^39^. Moreover, differences in ethnicity, dietary intake, exposure to environmental carcinogens and sample structure may be responsible for the discrepancies in the results. Earlier epidemiological studies have provided evidence that an interaction between alcohol consumption and C677T variation might contribute to modification of the risk for various types of cancer, such as head and neck, oesophageal, and colorectal cancers^40–45^. To explain the interactions of alcohol with the C677T genotype in previous studies, folate deficiency in mitochondria modified by alcohol exposure has been proposed to increase the risk of cancer^41,44^. This may induce an imbalance in DNA precursors leading to modified DNA synthesis and repair, which may contribute to the loss of normal control of proto-oncogene expression^40^. Other theoretical hypotheses also relate to the role of alcohol in folate metabolism and *MTHFR* polymorphism. For instance, alcohol might act as a folate antagonist to modify the function of folate receptors, which might lead to modified DNA synthesis or methylation, which has been proposed to be involved in the carcinogenesis process^7,11,46^. To the best of our knowledge, the underlying mechanisms explaining these interactions are not clearly established, and the findings from alcohol-gene interaction analyses must be interpreted with caution due to several considerations, such as reduced numbers of observations in subgroups and different categories for alcohol consumption in each study, along with different ethnicities^45^. Therefore, further studies at the molecular level are required to conclusively understand the relationship between alcohol and folate mechanism.

There are several strengths of this study. First, the study participants, consisting of case and control groups, were individually matched by age and sex. The use of matching and conditional logistic regression was advantageous for optimizing a case-control study (OR to yield increases in efficiency). Moreover, the study population was relatively larger than those in previous studies exploring the interactive effects between alcohol intake and *MTHFR* polymorphisms. From this perspective, the present investigation was the first novel study to suggest an interactive effect, with meaningful results that were obtained after adjustment for diverse covariates such as BMI, education, smoking status, and family history of thyroid cancer. By contrast, there were certain limitations in this study, mostly related to the limited number of subjects whose genetic information was available. We focused on analysing the data based on the status of alcohol experience (ever/never) to minimize any recall bias that may have occurred if average alcohol consumption was used. Furthermore, due to limited information, histopathological types were available only for 326 of the total 642 cases. Among them, 315 (96.6%) were PTC cases. However, when the subgroup analysis was performed for only PTC cases, the interaction between MTHFR C677T polymorphism and alcohol consumption was still significant (*P* = 0.012, table not shown).

In conclusion, our study supports an inverse association of alcohol consumption and an association between *MTHFR* polymorphisms and thyroid cancer risk. Additionally, among C/C homozygotes of the *MTHFR* C677T polymorphism, ever drinkers showed a suggestive protective effect on thyroid cancer risk, whereas this effect was not observed in individuals with a T+ allele. As there are no sound explanations for the protective effect of alcohol on thyroid cancer risk, it is important to further explore whether this relationship is due to an interaction or is a proxy for other risk factors, which was significantly observed for *MTHFR* C677T polymorphism in this study.

## Subjects and Methods

### Study population

**Figure 1:**
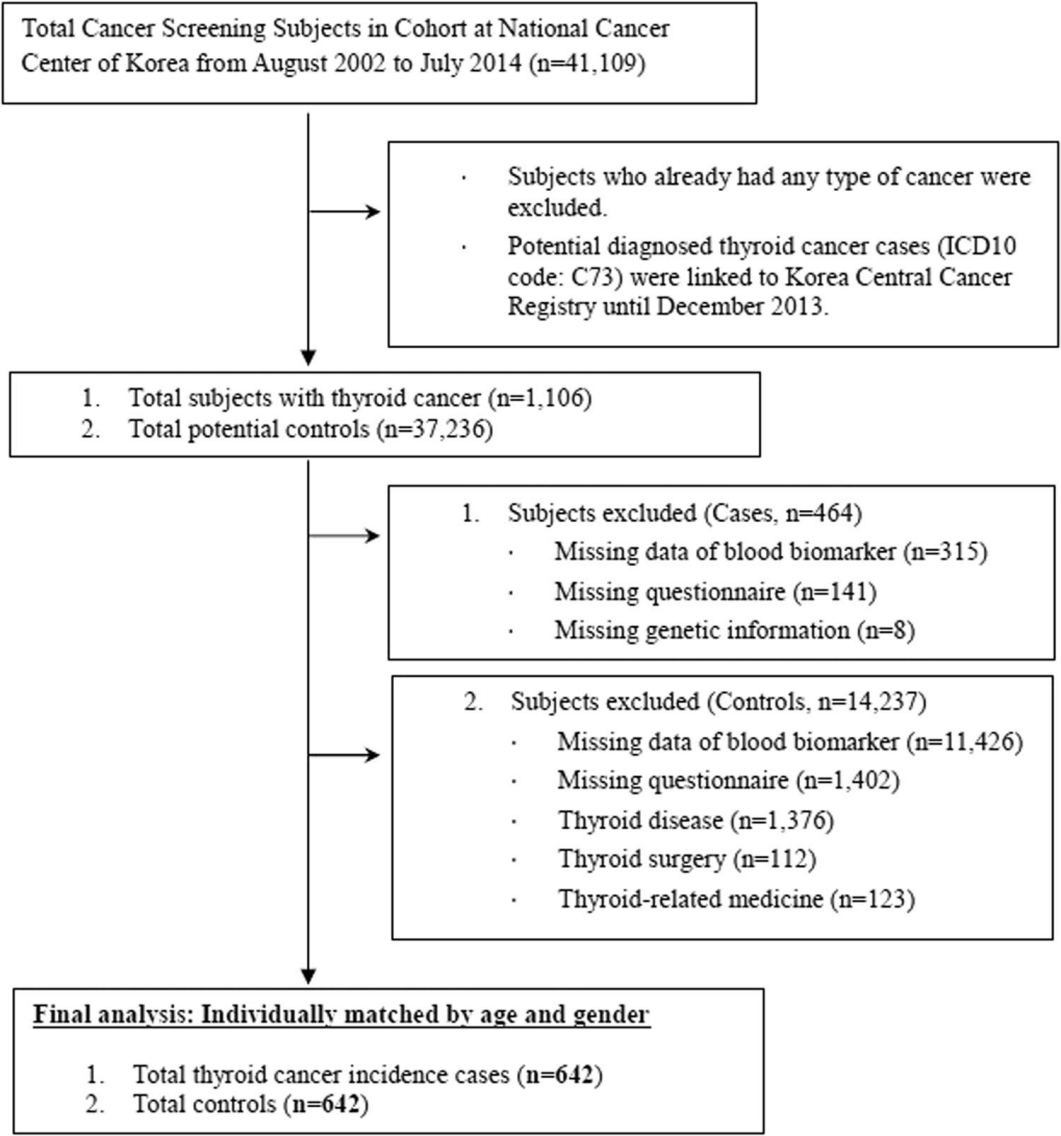
Flowchart of study subject selection.

The subjects of this hospital based case-control study were selected from the participants of the Cancer Screening Program at the National Cancer Center (NCC) of South Korea^47^ between August 2002 and July 2014 with a total of 41,109 individuals. Participants were individuals 30 years or older, who completed health-screening examinations as well as screening for selected cancers. At the baseline evaluation, the participants were asked to complete a self-administered questionnaire, and information on demographic characteristics, medical history, and lifestyle factors was collected. Subjects diagnosed with thyroid cancer (ICD10 code C73) were identified by linking to the Korea Central Cancer Registry (KCCR) database. From the subjects diagnosed with thyroid cancer (n = 1,106), individuals with missing questionnaires and blood biomarkers were excluded. Additionally, of the potential controls (n = 37,236), participants with a missing questionnaire or blood biomarker; those with a history of thyroid-related disease, surgery, or medicine usage; and those with a diagnosis of any type of cancer were excluded from the study. For the final analysis, 642 thyroid cancer cases (201 males, 441 females) and 642 individually age-and-sex matched controls (201 males, 441 females) were selected (Fig. 1). All of the participants in this study provided written informed consent, and the study protocol was approved by the Institutional Review Board of the NCC (#NCC2016-0088). All actual procedures utilized in the present study were performed in accordance with the guidelines and regulations of the IRB of the National Cancer Center.

### Alcohol intake measurement

Information on alcohol consumption behaviours was obtained from all participating individuals. The questionnaire asked whether the participants had a history of ever consuming alcoholic beverages regularly. Additionally, they were asked to provide information on the average frequency of drinking occasions, types of alcoholic beverages consumed (i.e., beer, soju, rice wine, wine, and spirits), the average number of drinks consumed on each drinking occasion, and the serving size of each alcoholic drink from a previous year. The average total daily alcohol intake (g/day) was calculated by summing the beverage-specific alcohol consumed using the provided information. The study subjects were classified as a never drinker, light drinker if their average daily intake of alcohol was less than 4.7 g/day (which is the median level of alcohol intake for all drinkers), or moderate or heavy drinker if their average alcohol intake exceeded 4.7 g/day.

### Genotype measurement

The study participants’ genomic DNA was extracted from peripheral blood leukocytes isolated from the obtained whole-blood samples. Genotyping was performed using the Infinium OncoArray-500K BeadChip (Illumina Inc., San Diego, California) with 533,631 genetic variants. After quality controlling for monomorphic variants, a minor allele frequency (MAF) < 0.01, call rate <95%, and deviation from Hardy-Weinberg equilibrium (*P*-value < 1 × 10^−6^), 345,675 single nucleotide polymorphisms (SNPs) were available for further analysis. The genetic variants C677T (rs1801133) and A1298C (rs1801131) of the *MTHFR* gene, which passed the above quality-control criteria, were selected for the analyses.

### Statistical analyses

General characteristics of the participants of this study were compared between thyroid cancer cases and controls using chi-square tests for categorical factors and Student’s t-tests for continuous variables. For the association analysis between alcohol consumption status, the *MTHFR* gene, and thyroid cancer risk, ORs and 95% CIs were calculated using conditional logistic regression for age-and-sex matched cases and controls. The model was further adjusted for BMI, smoking status, education level, and family history of thyroid cancer. Only the individuals with no missing data of confounders were included in the fully adjusted model. To evaluate the interaction between alcohol consumption and *MTHFR* SNPs, the likelihood ratio test, where the multiplicative term of alcohol consumption and the SNPs was inserted to the logistic regression model, was performed. The datasets generated and/or analysed during the current study are available from the corresponding author upon reasonable request.

## Electronic supplementary material

Supplementary Information
