# Supplementary material for: Interaction between alcohol consumption and methylenetetrahydrofolate reductase polymorphisms in thyroid cancer risk: National Cancer Center cohort in Korea
Source: Sci Rep. 2018 Mar 6;8:4077. doi: 10.1038/s41598-018-22189-w (PMC5840348; doi:10.1038/s41598-018-22189-w)
Supplement: Supplementary file 1 — Supplementary Information [file 41598_2018_22189_MOESM1_ESM.pdf]

Supplementary Information:

Interaction between alcohol consumption and methylenetetrahydrofolate reductase polymorphisms in thyroid cancer risk: National Cancer Center cohort in Korea

Sarah Yang[1,2], Jeonghee Lee[1], Yoon Park[1], Eun Kyung Lee[3], Yul Hwangbo[3], Junsun Ryu[3], Joohon Sung[2], and Jeongseon Kim[1]*

[1] Molecular Epidemiology Branch, Division of Cancer Epidemiology and Prevention, Research Institute, National Cancer Center, South Korea. [2]Complex Disease & Genomic Epidemiology Branch, Department of Public Health, School of Public Health, Seoul National University, South Korea. [3]Center for Thyroid Cancer, National Cancer Center Hospital, National Cancer Center, South Korea

*Corresponding Author: Jeongseon Kim (e-mail: jskim@ncc.re.kr)

SUPPLEMENTAL TABLE 1

Association between alcohol consumption by dose and thyroid cancer risk, stratified by sex[1]

	Control	Case	Crude OR (95% CI)	Fully adjusted OR (95% CI)[2]
**Total (*n* = 1130)**				
Alcohol drinking status by dose [*n* (%)]				
Never drinker	263(45.34)	289(52.55)	1.00	1.00
Light drinker (<4.7 g/day)[3]	151(26.03)	119(21.64)	0.68(0.50-0.93)	0.71(0.51-0.98)
Moderate/heavy drinker (≥4.7 g/day)[3]	166(28.62)	142(25.82)	0.67(0.46-0.96)	0.71(0.48-1.03)
**Males (*n* = 336)**				
Alcohol drinking status by dose [*n* (%)]				
Never drinker	24(14.12)	32(19.28)	1.00	1.00
Light drinker (<4.7 g/day)[3]	33(19.41)	26(15.66)	0.51(0.24-1.09)	0.60(0.26-1.36)
Moderate/heavy drinker (≥4.7 g/day)[3]	113(66.47)	108(65.06)	0.73(0.40-1.31)	0.80(0.43-1.49)
**Females (*n* = 794)**				
Alcohol drinking status by dose [*n* (%)]				
Never drinker	239(58.29)	257(66.93)	1.00	1.00
Light drinker (<4.7 g/day)[3]	118(28.78)	93(24.22)	0.73(0.52-1.02)	0.75(0.53-1.07)
Moderate/heavy drinker (≥4.7 g/day)[3]	53(12.93)	34(8.85)	0.55(0.33-0.92)	0.58(0.34-1.00)

[1] OR, odds ratio; CI, confidence interval; estimates of OR and 95% CI were all rounded to the nearest tenth.

[2] Adjusted by body mass index, smoking status, education level, and 1st degree family history of thyroid cancer.

[3] 50 percentile (median) of alcohol intake.

SUPPLEMENTAL TABLE 2

Characteristics of *MTHFR* variants in the study

	Physical position	Type	Minor/major allele	Minor allele frequency	Linkage Disequilibrium (R^2)
Genetic polymorphisms of *MTHFR* on Chromosome 1					
A1298C (rs1801131)	11854476	Missense	C/A	0.17	0.17
C677T (rs1801133)	11856378	Downstream	T/C	0.46	
